# The cAMP Pathway as Therapeutic Target in Autoimmune and Inflammatory Diseases

**DOI:** 10.3389/fimmu.2016.00123

**Published:** 2016-03-31

**Authors:** Verena Katharina Raker, Christian Becker, Kerstin Steinbrink

**Affiliations:** ^1^Department of Dermatology, University Medical Center Mainz, Johannes Gutenberg-University Mainz, Mainz, Germany

**Keywords:** cyclic AMP, autoimmunity, targeted therapies, inflammation, T cells, Tregs, T regulatory cells

## Abstract

Nucleotide signaling molecules contribute to the regulation of cellular pathways. In the immune system, cyclic adenosine monophosphate (cAMP) is well established as a potent regulator of innate and adaptive immune cell functions. Therapeutic strategies to interrupt or enhance cAMP generation or effects have immunoregulatory potential in autoimmune and inflammatory disorders. Here, we provide an overview of the cyclic AMP axis and its role as a regulator of immune functions and discuss the clinical and translational relevance of interventions with these processes.

## Introduction

Cells must be able to sense and integrate countless extracellular and intracellular signals and adapt their cellular functions. Second messengers serve as initiating components of intracellular signal transduction cascades that transmit signals by cellular messengers that depend on extracellular signaling molecules (1). Thereby second messengers serve to greatly amplify the strength of the original first signal. Cyclic adenosine monophosphate (cAMP) was the first discovered intracellular second messenger of extracellular ligand action (2). Within the immune system, cAMP regulates pro- and anti-inflammatory activities: drugs that elevate intracellular cAMP levels reduce the production of pro-inflammatory mediators and increase the production of anti-inflammatory factors in numerous immune cells. This review aims to shed light on the variety of processes influenced by cAMP in the immune system with regard to treatment options in diseases.

## The cAMP Pathway

Cyclic adenosine monophosphate, identified in 1957 (2) as the first intracellular second messenger of extracellular ligand action, is now established as a universal regulator of metabolism and gene expression in all life forms (3). A family of enzymes called adenylate cyclases (AC) catalyzes cAMP formation from ATP. In vertebrates, AC comprise nine membrane-bound isoforms and one soluble isoform (4). AC vary in distribution and developmental expression and their regulation is complex and isozyme specific. In addition to AC expression and activity cAMP homeostasis is regulated by a superfamily of phosphodiesterases (PDE) that degrade intracellular cyclic nucleotides. PDE comprise more than 100 enzyme variants divided into 11 families (5) based on their structure, specificity for, and modulation by, cyclic nucleotides. Certain PDE increase their activities in response to cAMP and cAMP stimulates the synthesis of new PDE mRNA (6, 7), resulting in a feedback loop between cAMP levels and PDE activity. Contributing to the complexity of the pathway, some PDE families are strictly cAMP-specific (PDE 4, 7, and 8), whereas others are cyclic guanosine monophosphate (cGMP)-specific (PDE 5, 6, and 9) (8, 9). Additional families hydrolyze both cAMP and cGMP (PDE 1, 2, 3, 10, and 11), establishing cross-regulation of both pathways with important implications in the utility of pharmacotherapeutic agents targeting cyclic nucleotide metabolism (10, 11).

**Figure 1:**
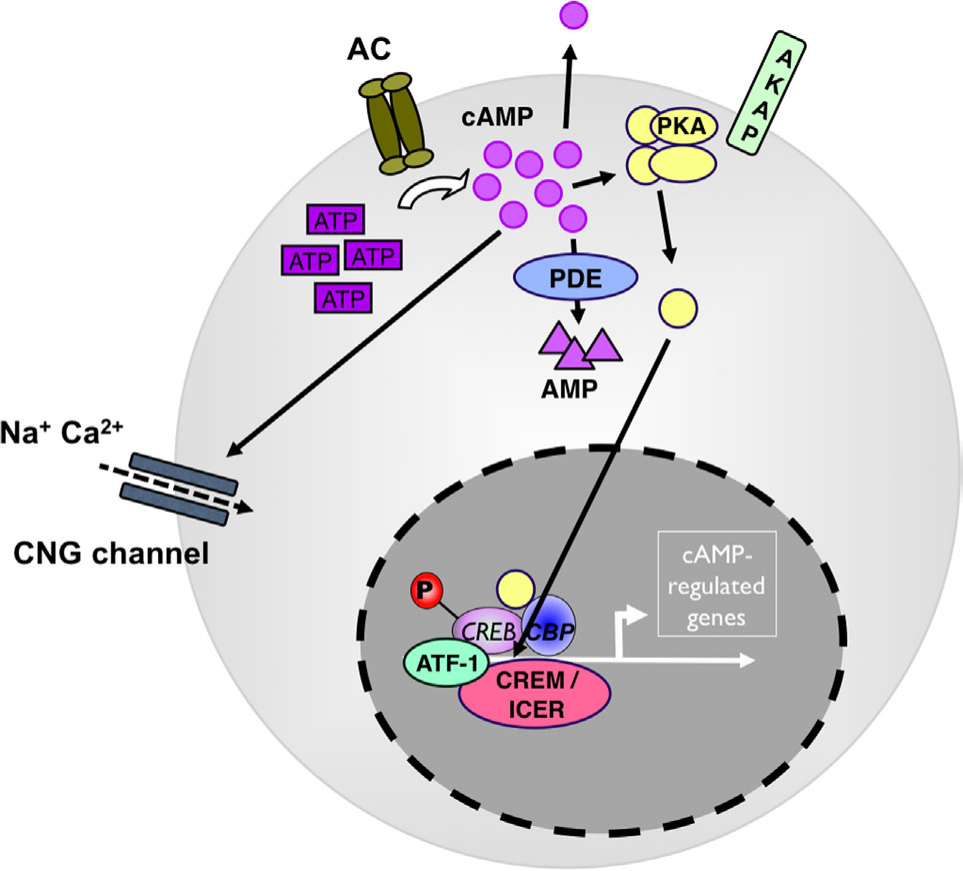
**cAMP as a regulator of immunity**. Adenylate cyclases (AC) produce cAMP from adenosin-tri-phosphate (ATP). High levels of cytosolic cAMP lead to activation of protein kinase A (PKA). PKA stimulation induces the phosphorylation of transcription factors, such as CREB, ICER/CREM, ATF-1, and CBP to drive camp-driven genes. Phosphodiesterase 4 (PDE4) decreases intracellular cAMP levels and counterbalances the intracellular cAMP effect. ATF, cAMP-dependent transcription factor; CBP, cAMP-binding protein; CNG, cyclic nucleotide-gated ion channel; CREB, cAMP response element-binding protein; ICER, inducible cAMP early repressor; P, phosphorylation.

As a second messenger, cyclic AMP serves in multiple downstream pathways. Most prominent, it activates the cAMP-dependent protein kinase A I (PKA) (12) (see Figure 1). Upon binding of cAMP to the regulatory subunits, PKA dissociates into its regulatory and catalytic subunits and the catalytic subunits phosphorylate specific *Ser* and *Thr* residues on numerous target proteins initiating successive signaling cascades, particularly in nutrient metabolism (13). In addition, cAMP-activated PKA binds and phosphorylates cAMP-responsive transcription factors, including cAMP-response element binding protein (CREB), members of the cAMP-responsive element modulator/inducible cAMP early repressor (CREM/ICER) protein family (14), activating transcription factor-1 (ATF-1), NFκB, and nuclear receptors (see Figure 1). Phosphorylated CREB, CREM, and ATF-1 interact with the transcriptional coactivators CREB-binding protein (CBP) and p300 when bound to cAMP-response elements (CREs) in target genes (15). In addition to PKA activation, cAMP also directly modulates the activity of guanine-nucleotide-exchange factor (GEF) exchange proteins (Epacs) and cyclic nucleotide-gated channels (CNGs) (16) all with important roles in cellular functions (17, 18). In addition to PKA, CREB, CREM, and ATF-1 can all be phosphorylated by many other kinases, and the action of PKA is counterbalanced by specific protein phosphatases.

Basal cytosolic cAMP levels are in the low micrometer range (19). In the cytosol, cAMP is not evenly distributed but rather forms submembranous spatially discrete pools generated in microdomains containing AC, PDE next to PKA localized by A-kinase-anchoring proteins (AKAPs) (20). Specificity in cAMP signaling and fine and selective tuning of its different tasks is ensured by the differential expression of distinct isoforms and splice variants of anabolic, katabolic, and signaling molecules in various tissues and cell types and by differential composition of cAMP microdomains (21). Although various cAMP activities can have redundant, independent, or opposing effects within the same cell (22), some individual AC and PDE knockout and transgenic mice (23, 24) show specific phenotypes. In particular, individual PDE control select cyclic nucleotide-regulated events by being integrated into non-overlapping multi-molecular regulatory signaling complexes, suggesting cell or tissue-specific interference points (25, 26).

Eventually, an important, often overlooked aspect of the pathway consists in the secretion of cAMP into extracellular space and its transmission via gap junctions between cells (27). Whereas transmitted cAMP directly contributes to intracellular cAMP levels, excreted cAMP is converted into AMP and adenosine by cell surface bound PDE and ecto-5′-nucleotidases CD39 and CD73. By signaling through A2A and A2B adenosine receptors, extracellular adenosine stimulates AC and increases intracellular cAMP generation (28). Knockout mice with disrupted CD39 and CD73 have underscored the importance of the extracellular cAMP–adenosine feedback mechanism in physiological processes (29, 30). In the immune system extracellular cAMP may contribute to regulatory T cells (Treg) function (31, 32) and has been shown to promote monocyte differentiation into dendritic cells (DCs) (33).

## Cyclic AMP in Immune Homeostasis and Pathophysiology

**Figure 2:**
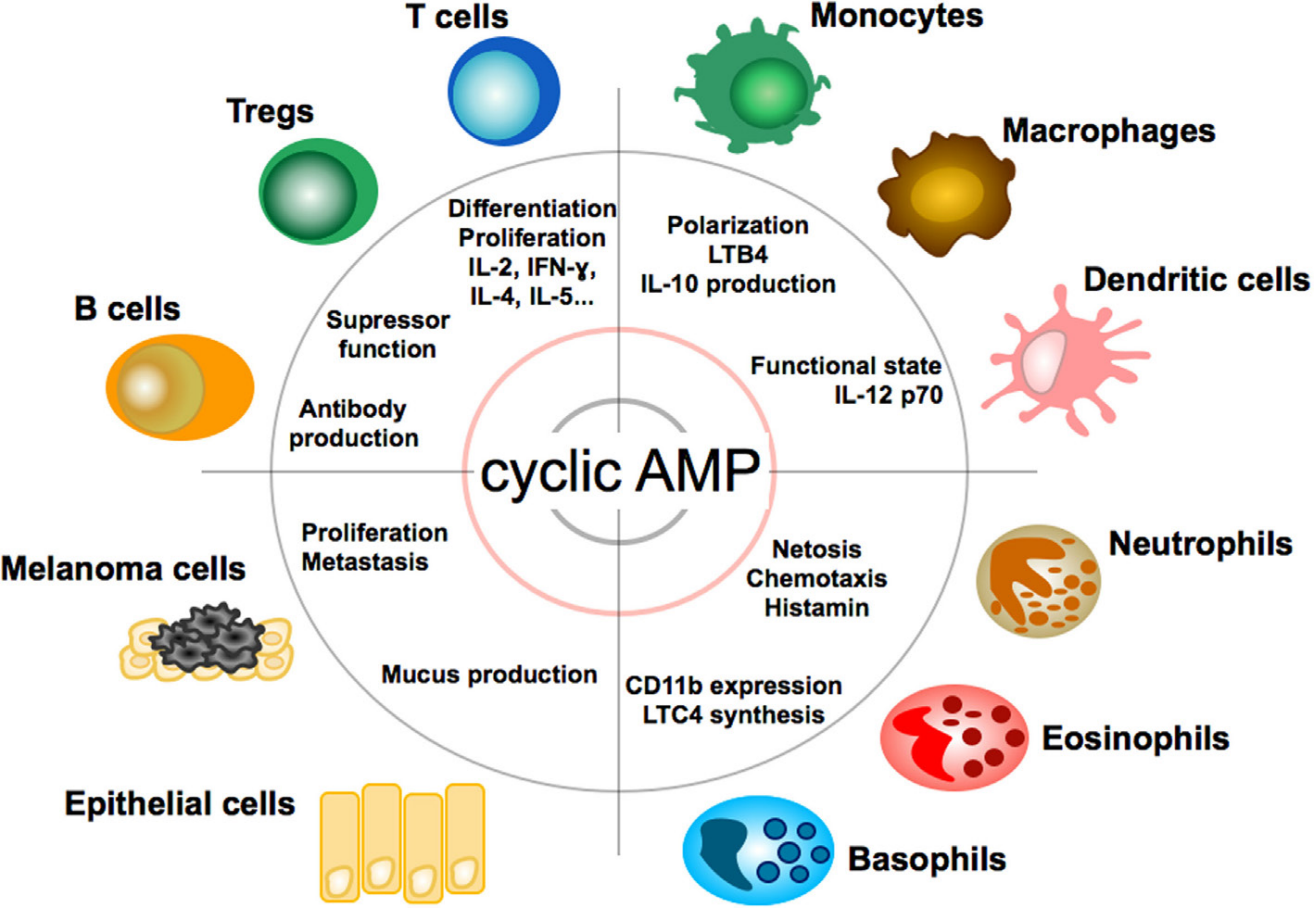
**Effect of cAMP on immune, tumor, and epithelial cells**. Impact and function of cyclic adenosin monophopshate (cAMP) on T and B lymphocytes, granulocytes, monocytes, macrophages, dendritic cells, epithelial cells, and melanoma cells. LTB4, leukotriene B4; LTC4, leukotriene C4.

Due to its multiple roles in cell physiology cAMP exerts broad modulatory effects on a variety of cells (see Figure 2). In the immune system, cyclic AMP regulates both innate and adaptive immune cell activities (34).

### Monocytes and Granulocytes

The functional state of monocytes orchestrates inflammatory and reparative phases in inflammatory responses and appears to be accompanied by changes in their intracellular cAMP levels. In the mouse, two major types of monocytes, Ly6C^high^ and Ly6C^low^, circulate in blood. Ly6C^high^ monocytes display pro-inflammatory activity, whereas Ly6C^low^ monocytes are patrolling cells, monitor tissue integrity, and exert anti-inflammatory and tissue repair activities (35). The orphan nuclear receptor Nr4a1 (Nur77) regulates the expression of genes linked to inflammation. Inflammatory stimuli inhibit its expression and induce an inflammatory Ly6C^high^ phenotype (36, 37). In turn, Nur77 is upregulated and represses numerous inflammatory genes in the transition from an inflammatory Ly6C^high^ to anti-inflammatory Ly6C^low/neg^ state (38–40). Elevated cAMP levels induce Nur77 expression (41) and, thus, favor a reparatory monocyte phenotype (42). Through these effects on phagocytes increased cAMP levels affect myeloid cell immunity against pathogen and parasites (43–45) and may also affect the differentiation of tumor-infiltrating myeloid-derived suppressor cells (MDSCs) by repression of TNF-α production. In regard of the latter CREB activation has been shown to upregulate miR-9 expression that promotes the differentiation of the so-called MDSCs with significantly increased immunosuppressive function (46).

In sum, increased cAMP levels appear to generally weaken monocyte inflammatory functions (47–50). Interestingly, bacteria and fungi have taken advantage of this effect in the course of evolution. Pathogen capture and programed destruction are among the most important activities of innate immune cells to prevent tissue invasion and pathogen dissemination. Certain microbacteria and fungi have evolved to hijack the host cAMP axis by introducing microbial adenylyl and guanylyl cyclases (51) and by intoxicating the host cell with preformed cAMP or adenylate cyclase toxins (52–54). Bordetella pertussis, for example, suppresses neutrophil extracellular trap (NET) formation by overwhelming leukocytes with supraphysiologic intracellular cAMP levels (55). Likewise, bacterial-derived or -induced cAMP facilitates intracellular bacterial survival by multiple actions, including CREB-dependent anti-apoptotic signaling and repression of intracellular bacterial killing in invaded monocytes and macrophages.

### NK Cells

Natural killer (NK) cells are capable of destroying tumor cells and virally infected cells (cytolysis) without prior sensitization. In NK cells, cAMP levels regulate target cell adherence and cytotoxic function. Both pharmacological repression and induction of cAMP inhibit perforin-mediated and CD95 ligand-mediated target cell lysis (56–60).

### Dendritic Cells

As professional antigen-presenting cells of the immune system, DCs are equipped with a unique capability to induce and regulate adaptive immune responses. In DC, cyclic AMP suppresses the release of pro-inflammatory mediators (TNF-α, IL-17, IFN-γ) (61) and promotes the release of anti-inflammatory mediators, such as IL-10 (62). As a functional consequence, cAMP concentrations in DC regulate T cell immunity (63). Pharmacological inhibition of cyclic nucleotide PDE4, which is highly expressed in DC, for example, suppresses the DC Th1-polarizing capacity (64, 65) and commands secretion of IL-6 and TGF-beta and subsequent induction of Th17 differentiation (66). It, thus, appears that cAMP levels differentially regulate cytokine production by DC as a response to changes in the microenvironment. Apart from spatio-temporal fine-tuning of DC activities, cAMP activities in DC depend on the stage of DC maturation: prostaglandin E2 (PGE2), a key inducer of cAMP, exerts a stimulatory function for immature DCs in peripheral tissues (67) but inhibitory function for mature DCs in lymph nodes (68).

### B and T Cells

In addition to innate cell function, cAMP also controls numerous adaptive immune cell activities. In adaptive immune cells, cAMP is essentially required in the induction of antigen-stimulated activation (69–72) but subsequently limits activation by negatively regulating signaling through B cell and T cell receptors (TCR). In B cells, it provides an essential signal in the induction of antigen-stimulated proliferation and antibody production (69, 70, 72). Elevation of intracellular cAMP enhances IgE production by promoting recombination of the Ig heavy chain loci and by favoring Th2 differentiation. In T cells, cAMP participates in the regulation of nearly all functional activities ranging from peripheral maintenance of naïve T cells (73) to their activation via the TCR (74), acquisition of effector function (75, 76), and memory (77). In cognate activation, cAMP acts as a temporary inhibitory feedback signal that limits T cell activation through the cAMP–PKA–Csk signaling pathway (74). Unlike temporary increases, continuously elevated cAMP levels induce an anergy-like state (78, 79). Likewise, anergizing TCR signals result in increased intracellular cAMP concentrations that upregulate the cyclin-dependent kinase (CDK) *inhibitor* p27kip1, sequester cyclin D2–cdk4, and cyclin E/cdk2 complexes and prevent progression through the G1 restriction point of the cell cycle (80). Furthermore, cAMP levels regulate the acquisition of effector function. Pharmacological upregulation of cAMP by inhibition of PDE activity, for example, prevents the development and function of cytotoxic T lymphocyte (CTL) (81). The significance of cAMP in acquisition of effector functions in T cells is also reflected by the observation that CREB mutant mice have normal T cell numbers in the thymus but exhibit a marked defect in peripheral T cell proliferation and IL-2 production, resulting from G1 cell-cycle arrest and apoptotic cell death (82). Most prominent, cAMP forms an essential component of the suppressive mechanism in Treg (83–92). Treg contain increased levels of cytosolic cAMP, further upregulate their cAMP level upon activation and consign cAMP to target cells via gap junctions (83, 85). In the target cell, cAMP inhibits the proliferation and differentiation of effector functions, in part by interfering with gene expression via ICER (90). Repression of cAMP accumulation in Treg by either adenylyl cyclase inhibition, application of a cAMP-specific antagonist, or PDE overexpression abrogates murine and human Treg suppression (83, 84, 86, 91, 93). Inversely, blockade of cAMP degradation by PDE inhibition improves Treg-mediated suppression in a murine asthma model (85). In line, non-functional Treg in Foxp3-mutant scurfy mice harbor significantly reduced levels of cytosolic cAMP (94).

**Figure 3:**
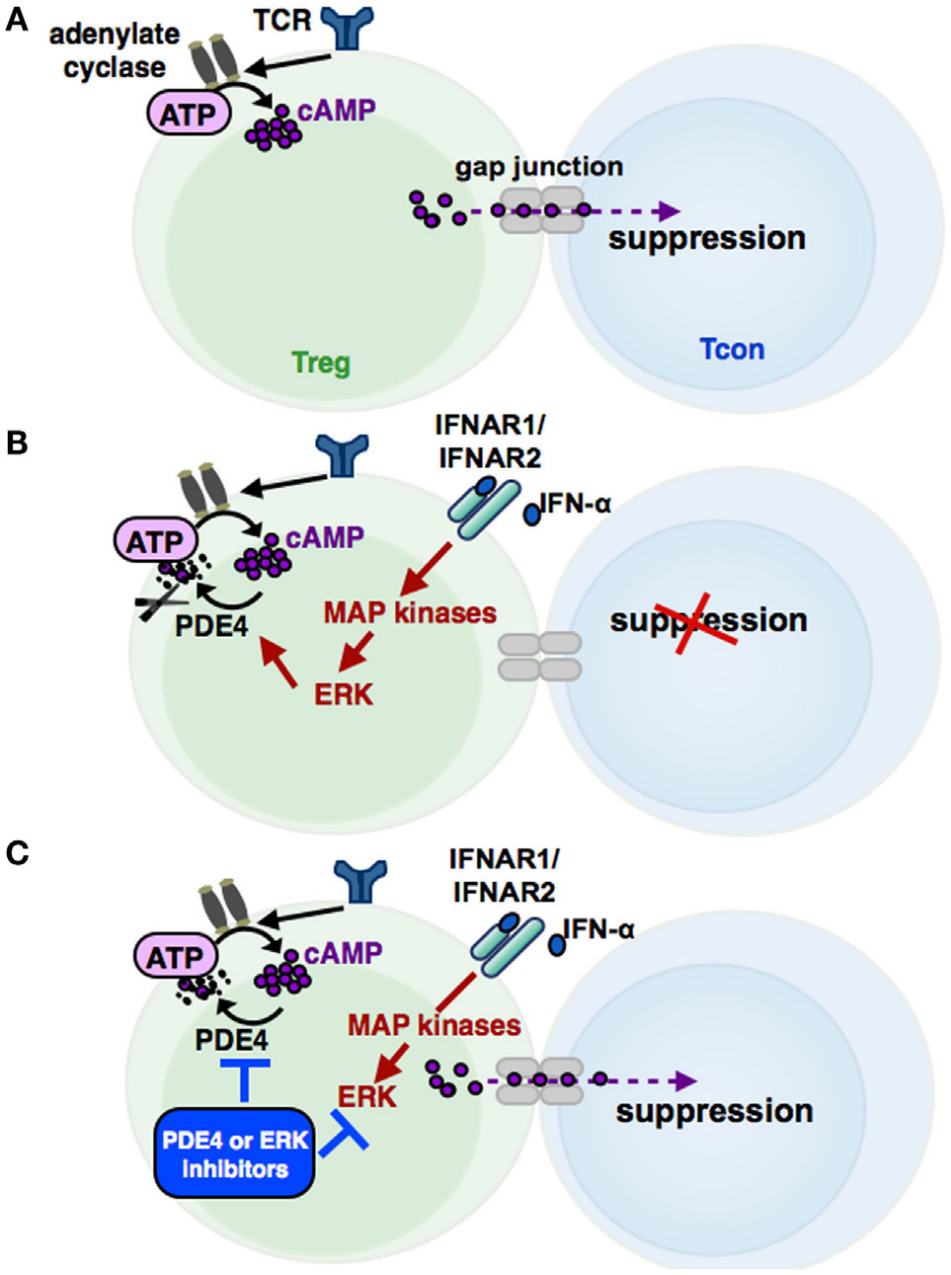
**The cAMP pathway in Treg and its regulation by IFN-α**. Signaling via the T cell receptor (TCR) leads to an activation of adenylate cyclases, resulting in high cAMP levels in regulatory T cells (Treg). cAMP can be transferred via gap junctions into conventional T cells (Tcon), thereby mediating the suppressive activity of Treg **(A)**. Phosphodiesterase 4 (PDE4), which can be activated by MAP kinase ERK-related pathways, reduces cAMP amounts in Treg by enzymatic cleavage, impairing the regulatory activity of Treg **(B)**. IFN-α abolishes the suppressive function of Treg by cAMP reduction, restoring the Tcon activation. Inhibition of the ERK or PDE4 pathway, respectively, results in a renewed suppressive capacity of IFN-α treated Treg **(C)**.

**Figure 4:**
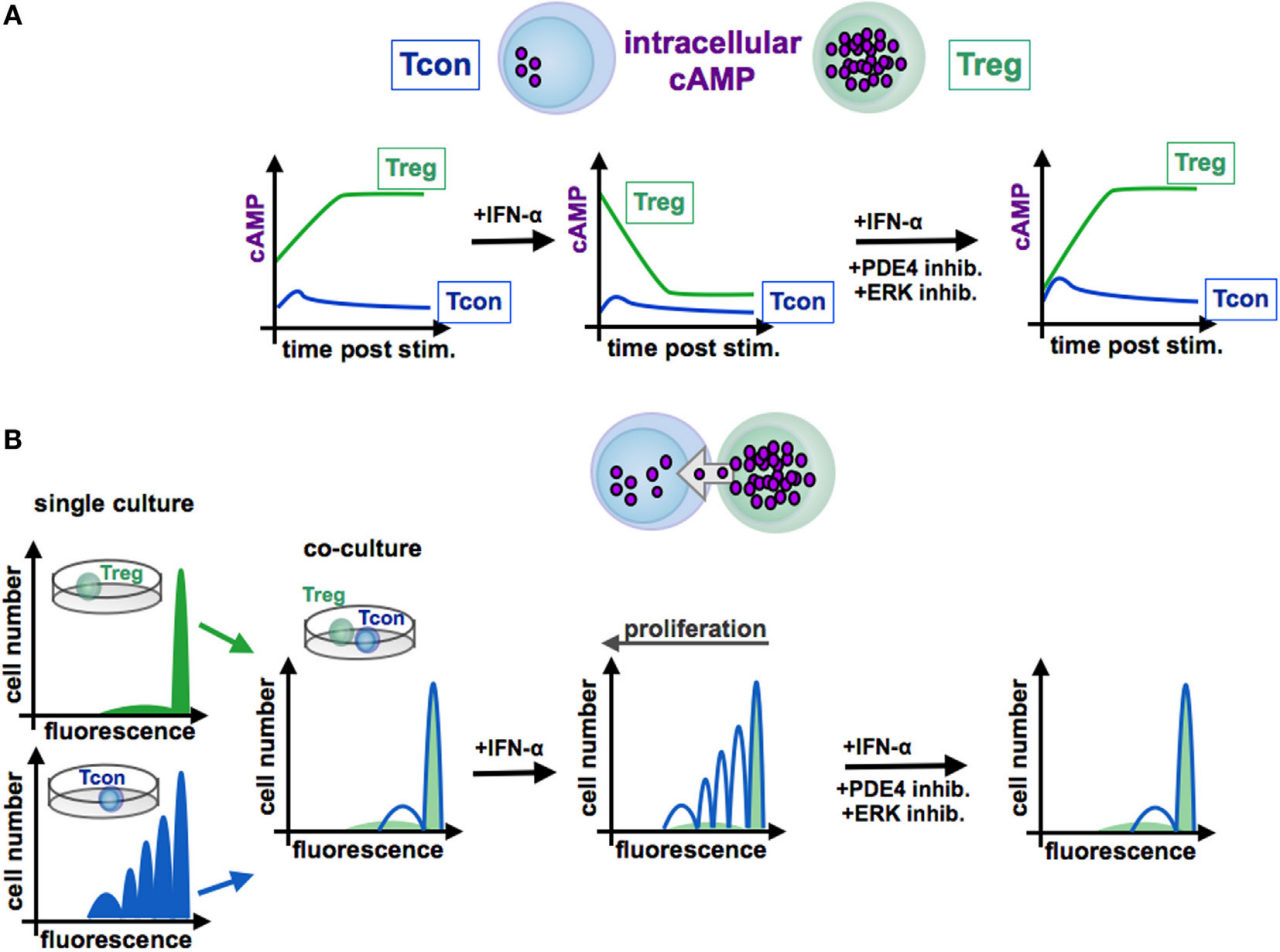
**Function of cAMP in the interaction of conventional and regulatory T cells**. In contrast to Tcon (blue line), Treg (green line) exhibit high levels of cAMP **(A)**. Stimulated Tcon display a high proliferation whereas Treg are characterized by a low proliferative capacity [**(B)**, left panels, single culture]. Treg efficiently inhibit Tcon proliferation in co-culture experiments by cAMP transfer via gap junctions to Tcon [**(B)**, co-culture]. By contrast, IFN-α abrogates the suppressive function of Treg through reduction of cAMP levels [**(A)**, centered panel], resulting in a restored Tcon activation [**(B)**, centered panel]. Blockade of the ERK or PDE4 pathway, respectively, increases intracellular cAMP amounts [**(A)**, right panel], renews the suppressive activity of Treg [**(B)**, right panel]. Tcon, conventional T cells; Treg, regulatory T cells.

Increased cAMP formation in Treg is a prerequisite for their suppressive activity (95) (see Figures 3 and 4). Constitutively high cAMP levels in Treg appear to be caused by Foxp3-induced decreased PDE3B expression (96) and increased AC9 activity (87) driven by their constitutive active state (95). During Treg-mediated suppression, cAMP is transferred via gap junctions to conventional T cells (Tcon), where it represses IL-2 production and inhibits the proliferative response (83). Pharmacological inhibition of cAMP formation abrogates the suppressive function of Treg (see Figure 3) (91).

In this context, Bacher et al. showed that IFN-α, an antineoplastic agent with well-known autoimmune side effects, disturbs the immunosuppressive activity of human CD4^+^CD25^+^Foxp3^+^ Treg by disabling cAMP upregulation upon activation (92, 97) (see Figure 3 and 4). IFN-α-mediated inhibition of Treg suppression can be partially restored by pharmacological inhibitors blocking ERK and PDE/PDE4 activity through specific inhibitors (92, 97) (see Figures 3 and 4). These results are in line with the observation that human T cells predominantly express the short PDE4B and PDE4D isoforms, functionally regulated by the ERK2 MAP kinase (98, 99). As PDE have an essential role in the IFN-α-mediated inhibition of Treg, PDE4 interference by specific inhibitors may represent a therapeutic option to restore immune regulation in autoimmune diseases, such as psoriasis or lupus erythematosus, accompanied by reduced Treg function (64, 100).

Next to its role in the Treg-suppressive mechanism cAMP is required for the generation and maintenance of Treg: the cAMP-responsive transcription factor CREB stabilizes FoxP3 expression and promotes and maintains the Treg phenotype (101, 102). Treg essentially depend on IL-2 for their peripheral maintenance and suppressive activity (103, 104) and their number and activity can be therapeutically manipulated by low-dose IL-2 and particular IL-2/anti-IL-2 complexes (105, 106) to control autoimmune diseases and inflammation (107). Interestingly, IL-2 may contribute to increased cAMP production in Treg by increasing adenylate cyclase AC7 activity (88). In conjunction with its role in control of the Treg phenotype, its transmission via gap junctions to and from Treg also appears to play a role in the Treg lifecycle as evidence by the observation that Treg numbers are significantly reduced in connexin 43 knockout mice (108).

Some viruses prevent their rejection by the immune system by interfering with the cAMP pathway in T cells. HIV-1 surface glycoprotein gp120 induces anergy in naive T lymphocytes (109, 110) and increases cAMP levels and suppressive activity in Treg (86, 111, 112). In turn, cAMP repression restores antiviral T cell function in HIV patients (113).

Beyond their role in immune regulation, Treg take on homeostatic functions by regulating metabolic activity in visceral fat and participating in tissue repair. Functionally distinct Treg accumulate in injured skeletal muscle and contribute to repair processes. Muscle Treg distinctly express the growth factor amphiregulin, which improves muscle repair by directly acting on muscle satellite cells (114). In line with outlined role of cAMP in Treg function, amphiregulin synthesis is inhibited by PKA inhibitors and enhanced by ligands that increased cAMP or directly activate the PKA (115).

Together these findings classify cAMP as a key component of immune cell function and disclose cAMP-regulating enzymes as molecular targets for therapeutic intervention with immune activities in pathological processes like allergy and autoimmunity.

## Modulation of cAMP in Autoimmune and Inflammatory Diseases

Cyclic AMP is a central player in the network of signaling pathways underlying pathogenesis of several diseases and several interference points are used therapeutically in a variety of conditions. Although the clinical impact of changes in cAMP remains incompletely defined, one fundamental conclusion can nevertheless be drawn: interventions that enhance cAMP generation or actions have immune dampening potential; conversely, repression of cAMP or cAMP signaling has immunostimulatory capability.

Formation of cAMP by AC and degradation by PDE identifies AC and PDE as major targets for therapeutic intervention with cAMP levels. To date, the AC activity has been mostly pharmacologically targeted through agonists or antagonists ­affecting upstream G-protein-coupled receptors (GPCR) (23, 116). However, AC knockout and transgenic mice revealed individual and clearly distinct physiological functions for AC isoforms (23). The observation that individual isoforms play a dominant role in specific tissues has led to AC being considered as main drug targets (117). In order to achieve selective interference, isoform-selective compounds are required. Such compounds are currently being sought and tested. Here, the idea is pursued, that selective inhibitors intervene in a tissue-specific manner, but remain ineffective in tissues that express various AC isoforms (118).

AC-specific compounds already reached preclinical stages and others have been approved for particular diseases, such as colforsin daropate hydrochloride (NKH447), a AC5 selective forskolin (FSK) derivate, for the treatment of advanced congestive heart failure (119, 120). Thus, even though AC isoform-targeted drugs are still in early stages of the development, the finding that AC have clearly separated physiological functions at least suggests AC as pharmacologic targets in a broad spectrum of diseases ranging from neurodegenerative disorders to congestive heart failure and lung diseases as asthma and chronic obstructive pulmonary disease (COPD).

Since their identification in 1958 (2), continuing efforts have been undertaken to advance the understanding of PDE biology and function, and PDE have been considered pharmacological targets in various diseases, such as pulmonary diseases like COPD and asthma, depression, schizophrenia, erectile dysfunction, and autoimmune disease like psoriasis/psoriasis arthritis and rheumatoid arthritis (8, 100, 121–125). Although numerous PDE inhibitors have been developed, their introduction into the clinic has been hampered by their narrow therapeutic window and side effects, such as nausea and emesis, occurring even at sub-therapeutic levels.

In the immune system, PDE family 3, 4, and 7 members represent the predominant cAMP-degrading enzymes (126). PDE4 are encoded by four separate genes (*PDE4A–D*) and each PDE4 controls non-redundant cellular function (127). In addition, more than 20 PDE4 variants arise from alternative mRNA splicing or the use of different transcriptional units (5). While PDE4A, PDE4B, and PDE4D are expressed in immune cells (T and B cells, neutrophils, eosinophils, DCs, monocytes, macrophages), PD4C is minimally active or absent (128, 129). PDE3 and PDE7 are detected in most inflammatory cells, including T and B cells, NK, and myeloid cells (6, 59, 127, 130–132). However, PDE4s are the predominant cAMP-degrading isoenzymes (126, 127). In addition, the expression levels of the PDE isoenzymes are differentially regulated by a variety of inflammatory stimuli (126, 127). Apart from immune cells, PDE4 members are also expressed in chondrocytes, smooth muscle cells, epithelial cells, and vascular endothelium (127). By increasing levels of intracellular cAMP, PDE4 inhibitors show anti-inflammatory effects in almost all inflammatory and immune cells and are known to suppress a multitude of inflammatory responses, including proliferation, chemotaxis, phagocytosis, and release of pro-inflammatory mediators, such as cytokine and chemokines, reactive oxygen species, lipid mediators, and hydrolytic enzymes (34, 126, 129). Numerous selective PDE4 inhibitors have been patented and some of them have been evaluated in clinical trials, including diseases, such as asthma, COPD, atopic dermatitis, rheumatoid arthritis, and psoriasis/psoriasis arthritis. However, most of these compounds had to be discontinued because of narrow therapeutic windows. Doses needed for an efficient treatment could not be reached due to side effects, such as nausea, emesis, diarrhea, and abdominal pain being the most common. It has been hypothesized that adverse side effects of the PDE4 inhibitors are a result of their non-selectivity to all four PDE4 subtypes and PDE4 inhibition in non-target tissues at doses similar (or lower) than needed for therapeutic efficacy. It is postulated that blocking of PDE4D in non-target organs promotes emesis (133). In view of side effect profile of second-generation PDE4 inhibitors, new strategies for the design of active and non-emetic compounds have been employed to overcome the adverse effects and to improve therapeutic effects. In this context, despite highly conserved catalytic domains of PDE4 isoenzymes, PDE4 subtype-specific inhibitors have been generated. For example, potent PDE4B inhibitors with more than 100-fold selectivity over PDE4D have been synthesized (134, 135). Compared with the non-selective PDE4 inhibitor cilomilast (134), selective PDE4B inhibitors demonstrated a potent anti-inflammatory activity and significantly less gastrointestinal side effects. In order to circumvent side effects observed upon oral administration, inhalation (136) and topical application (137) of PDE4 inhibitors have been explored in the treatment of airway inflammation and inflammatory cutaneous diseases. Two phase studies conducted with a PDE4 inhibitor (AN2728) in psoriasis and atopic dermatitis patients showed promising results (138, 139). The interest for PDE4 anti-inflammatory activity arose from early studies with the prototypic PDE4 inhibitor, rolipram (140). However, although PDE4 inhibitors have been mostly developed to treat lung diseases, such as asthma or COPD, no compound has yet reached the market for asthma treatment. By contrast, the orally active PDE4 inhibitor roflumilast (Daliresp^®^, Forest Pharmaceuticals) has been approved for COPD by the European Medicines Agency in 2010 and the U.S. Food and Drug Administration in 2011 based on four clinical trials. These studies have shown that roflumilast improves lung function and reduces the frequency of COPD exacerbations in patients with chronic bronchitis symptoms (141–144). Although side effects were generally mild to moderate, nausea, diarrhea, weight loss, and headache were still reported (145). Despite these side effects, roflumilast received approval for COPD with severe air flow limitations, symptoms of chronic bronchitis, and a history of exacerbations in several countries (146, 147).

Another currently marketed oral PDE4 inhibitor is apremilast (Otezla^®^, Celgene Corporation) that has been approved by the EMA and FDA for psoriasis and psoriasis arthritis, two autoimmune diseases, characterized by chronic inflammation, tissue and organ involvement, and accelerated growth cycle of skin cells. Apremilast was developed based on the rolipram and roflumilast pharmacophore by coupling a series of phthalimide analogs in order to optimize its activity and to decrease side effects (148). The safety and efficacy of apremilast for the treatment of patients with plaque psoriasis and psoriasis arthritis were evaluated in numerous multicenter, randomized, double-blind, placebo-controlled clinical trials (ESTEEM-1 and -2 for psoriasis, PALACE-1, -2, and -3 for psoriasis arthritis) (149–152). In the two ESTEEM trials, apremilast reduced the severity and extent of moderate-to-severe plaque psoriasis (including nail, scalp, and palmoplantar manifestations) versus placebo in adults. Similarly, in three PALACE trials (PALACE 1–3), apremilast improved the signs and symptoms of psoriasis arthritis relative to placebo in adults with active disease despite treatment with conventional synthetic and/or biologic disease-modifying anti-rheumatic drugs. According to the published clinical trials, apremilast was well tolerated in all study groups analyzed. Throughout phase II and III trials, the most frequently reported side effects consisted of headache, nausea, diarrhea, emesis, and nasopharyngitis and upper respiratory tract infection under continued treatment. However, the studies showed that the gastrointestinal adverse effects usually subside within a month of therapy.

It is an interesting result of the clinical studies that improved inhibitor specificity does not prevent side effects. This result suggests that the same or overlapping cell populations caused both wanted and unwanted effects. In view of recent research results regarding the expression and activities of anabolic and catabolic cAMP enzymes in immune cells, the question arises whether particular PDE4 inhibitor effects are caused by alteration of immune cell functions. This question is underlined by the similarity of side effects in PDE4 inhibitor studies and some immunotherapeutic approaches. Unfortunately, effects in individual immune cell populations have not been considered in clinical studies with PDE inhibitors so far. For a better understanding of the underlying causes of wanted and unwanted effects, such studies appear urgently needed. Alongside their specificity, effective interference with the cAMP pathway through inhibitors depends on their mechanism of action. Basically, inhibitors may act reversibly or irreversibly. Irreversible inhibitors bind to enzymes through covalent bonds. Covalent inhibitors have many desirable features, including increased biochemical efficiency of target disruption, reduced sensitivity toward pharmacokinetic parameters and increased duration of action that outlasts the pharmacokinetics of the compound. Only few inhibitors of this type, however, exist for anabolic and catabolic cAMP enzymes with the common ADCY inhibitor MDL-12,330A, a cyclo-alkyllactamide derivative supposedly representing an exception (153). Most inhibitors are reversible, bind to enzyme through non-covalent bonds, and typically address the ATP-binding site or the catalytic portion. With non-covalent inhibitors, cells can quickly become insensitive by recovering enzyme activity. To increase their activity, however, inhibitors can be coupled to proteins that regulate protein expression. A favorable example exists in proteolytic targeting, such as the ubiquitin proteasome system (UPS) (154). Proteolytic targeting chimeric molecules, or PROTACS comprise a UPS recognition motif coupled to an inhibitor via a linker. While a first generation of PROTACs suffered from limited cell-permeability, the second generation has been improved by using a HIF1α peptide fragment as an E3 ubiquitin ligase recognition motif to increase permeability (155). Thus, in addition to the development of more specific inhibitors to achieve selective interference, their inhibitory activity may be improved through proteolytic targeting, particularly by preventing target cell resistance.

## Conclusion and Perspective

Because of its central importance as a universal regulator of metabolism and gene expression, systemic intervention of the cAMP metabolism is associated with numerous, sometimes considerable, side effects. Additionally or alternatively to the development of isoform-specific AC and PDE inhibitors, new methods need to be found by which these inhibitors may be delivered to tissues and cells specifically. Novel strategies may encompass the development of highly specific agents, new routes of delivery (cutaneous, inhalation) or the use of nanoparticles for tissue or even cell-specific drug delivery. Since cAMP signaling controls very different processes in different cells, a better understanding of the cAMP-mediated activities in particular cell types could help to pave the way to more specific interventions in cell function. Unlike anabolic and catabolic cAMP metabolism, very few drugs engage in signal transduction yet and, thus, the potential use of such actions remains unclear. Although known for over 60 years, the cAMP signaling still reveals new functional details. Therapeutic intervention of its activities, thus, requires further elucidation of its role in individual cell types and its entanglements with other signaling and metabolic pathways.

## Author Contributions

All authors listed, have made substantial, direct, and intellectual contribution to the work, and approved it for publication.

## Conflict of Interest Statement

The authors declare that the research was conducted in the absence of any commercial or financial relationships that could be construed as a potential conflict of interest.
